# Cognitive computer training in children with attention deficit hyperactivity disorder (ADHD) versus no intervention: study protocol for a randomized controlled trial

**DOI:** 10.1186/s13063-015-0975-8

**Published:** 2015-10-24

**Authors:** Aida Bikic, James F. Leckman, Jane Lindschou, Torben Ø. Christensen, Søren Dalsgaard

**Affiliations:** Institute of Clinical Research, University of Southern Denmark, Winsløwsparken 19, Odense, Denmark; Department of Child and Adolescent Mental Health Services, Psychiatric Hospital Region of Southern Denmark, Kresten Phillipsens Vej 15, Aabenraa, Denmark; Yale Child Study Centre, Yale University, 230 South Frontage Road, New Haven, USA; Copenhagen Trial Unit, Centre for Clinical Intervention Research, Rigshospitalet, Copenhagen University Hospital, Copenhagen, Denmark; Public Health and Quality Improvement, Central Denmark Region, Aarhus, Denmark; Department of Economics and Business, National Centre for Register-based Research, Aarhus University, Fuglesangs Allé 4, Aarhus, Denmark

**Keywords:** ADHD, Cognitive training, Cognitive remediation, Cognition, Computer training, Nonpharmacological treatment

## Abstract

**Background:**

Attention Deficit Hyperactivity Disorder (ADHD) is a common neurodevelopmental disorder characterized by symptoms of inattention and impulsivity and/or hyperactivity and a range of cognitive dysfunctions. Pharmacological treatment may be beneficial; however, many affected individuals continue to have difficulties with cognitive functions despite medical treatment, and up to 30 % do not respond to pharmacological treatment. Inadequate medical compliance and the long-term effects of treatment make it necessary to explore nonpharmacological and supplementary treatments for ADHD. Treatment of cognitive dysfunctions may prove particularly important because of the impact of these dysfunctions on the ability to cope with everyday life. Lately, several trials have shown promising results for cognitive computer training, often referred to as cognitive training, which focuses on particular parts of cognition, mostly on the working memory or attention but with poor generalization of training on other cognitive functions and functional outcome. Children with ADHD have a variety of cognitive dysfunctions, and it is important that cognitive training target multiple cognitive functions.

**Methods/Design:**

This multicenter randomized clinical superiority trial aims to investigate the effect of “ACTIVATE™,” a computer program designed to improve a range of cognitive skills and ADHD symptoms. A total of 122 children with ADHD, aged 6 to 13 years, will be randomized to an intervention or a control group. The intervention group will be asked to use ACTIVATE™ at home 40 minutes per day, 6 days per week for 8 weeks. Both intervention and control group will receive treatment as usual. Outcome measures will assess cognitive functions, symptoms, and behavioral and functional measures before and after the 8 weeks of training and in a 12- and 24-week follow-up.

**Discussion:**

Results of this trial will provide useful information on the effectiveness of computer training focusing on several cognitive functions. Cognitive training has the potential to reduce cognitive dysfunctions and to become a new treatment option, which can promote a more normal neural development in young children with ADHD and thus reduce cognitive dysfunctions and symptoms. This could help children with ADHD to perform better in everyday life and school.

**Trial registration:**

ClinicalTrials.gov: NCT01752530, date of registration: 10 December 2012

## Background

ADHD is one of the most prevalent psychiatric disorders in child and adolescent psychiatry, affecting approximately 5 % of school-aged children and adolescents [1–3]. ADHD is associated with poor academic performance, poor social functioning [4, 5], increased risk of drug abuse [6, 7], psychotic disorders [8, 9] and criminality [10, 11], as well as increased mortality [12]. The etiology of ADHD is still unknown, but there is evidence for a complex interaction between multiple genes and environmental factors [13]. Empirical studies have shown structural and functional abnormalities in the brain of individuals with ADHD [14, 15]. Additionally, the brain in children with ADHD is characterized by a cortical maturation delay in terms of the reaching the peak cortical thickness [16].

A wide range of cognitive functions is affected in ADHD, yet a specific ADHD cognitive profile has not been identified [17]. The cognitive impairments are very heterogenic in severity and the affected areas. Spatial working memory, impulse inhibition and vigilance are found to be the most impaired functions according to two large meta-analyses of observational studies comparing cognitive functions in patients with ADHD with healthy participants [18, 19]. Functions like inhibitory control, selective and sustained attention, attention switching and processing speed are also significantly impaired [16, 20–22]. These features are associated with the executive control system [23–26] and are often manifest in early childhood and persistent over time [11, 27]. Executive dysfunctions are often seen in individuals with ADHD. Children with ADHD display significant impairment in executive functions compared to typically developing controls as a group, but only 50 % of the patients exhibit executive dysfunctions at the individual level [28].

Although there is some evidence supporting the beneficial effects of stimulant medication for ADHD [29], the treatment is not a cure as the symptoms return immediately after treatment discontinuation. Furthermore, 20 % to 30 % of individuals with ADHD do not show a positive response to stimulant medications, and long-term effects are variable [30–32].

### Cognitive training

Cognitive training is rooted in cognitive rehabilitation, based on the concept that direct training can result in a reorganization of neural functions. Among other effects, neuroplasticity allows the central nervous system to learn new skills, remember information and reorganize neural networks in response to external stimulation [33]. The basic mechanisms involved are neurogenesis, programmed cell death and activity-dependent synaptic plasticity [33]. Childhood is a period of changes in the brain’s anatomical structure and synaptic connections. A child’s brain is more susceptible to the environmental impact than the adult’s brain due to increased plasticity [33]. Thus, injuries and some neurological diseases are overcome by children faster and easier than by adults. Several studies indicate that the peak of brain plasticity is reached within the first 7 years of life [34], although the potential is likely to be lifelong. For example, a functional magnetic resonance imaging (fMRI) open trial of young healthy adults found that training working memory resulted in an increased brain activity in the dorsolateral, prefrontal, and parietal association cortex, indicating plasticity of the neural system [35]. These cortical areas are overlapping the prefrontal regions, which are likely implicated in the pathology of ADHD [36, 37]. Despite the hypothesis that children under the age of 7 have better neuroplasticity and therefore may benefit more from cognitive training as compared to older children, we have not identified any studies investigating the effect of cognitive training in different age groups.

Cognitive training is typically delivered in a computerized format and is aimed at training cognitive functions that are deficient in a patient population by using a special kind of computer games. A rapidly growing number of randomized trials support the hypothesis that cognitive dysfunctions can be trained in children with ADHD [38–41]. Most ADHD trials with children have focused solely on working memory training and findings have been somewhat inconsistent [42–44]. Overall, working memory training shows effects on verbal and spatial working memory [45], and these effects are generalized to improved sustained attention up to 6 months follow-up [42]. Some few studies have shown improvements in academic abilities, but there is no consensus yet as several newer studies had negative results [46–48]. Working memory, combined with response inhibition training, has shown significant improvements on symptoms, spatial working memory, ability to ignore distracting stimuli and sustained attention as rated by a significant other [41]. Training of executive functions improved parent-rated executive functions and ADHD behavior when compared to waiting-list condition [49].

Fewer trials have focused on the attention training in children with ADHD that results in a significant improvement in trained and untrained attention and vigilance [39, 50–52], a measure of school performance and a significant reduction in parent and teacher observation of inattention [39, 40, 53]. In addition, the effects on inhibition and working memory have been found [52], and significant changes in inattentiveness, behavior and executive functions measured by parent ratings on Behavior Rating Inventory of Executive Functions (BRIEF) [54].

Structural and functional correlates of cognitive training have been shown in several small studies. Enhanced activity in neural structures closely related to ADHD pathology [55] and increase of focal volumetric gray area in bilateral middle frontal cortex and right inferior-posterior cerebellum after attention and executive functions training [56]. Cognitive training has also been shown to induce neurochemical changes at the synapse in dopamine function after training [57].

In conclusion, randomized trials and observational studies suggest that cognitive training of children with ADHD has some beneficial effects. However, the empirical evidence in this field is still insufficient as most trials have a high risk of systematic errors (bias) mainly due to lack of blinding, incomplete outcome data, and selective outcome reporting. Further, most trials have small sample sizes, which result in an increased risk of imprecision. As children with ADHD have impairments in many different cognitive functions, there is a need for randomized trials to examine effects of broader cognitive training, rather than focusing on only one or two domains, for instance, working memory, response inhibition or sustained attention. It is important to validate and extend existing knowledge on the effects of cognitive training for patients with ADHD.

Hence, to overcome some of these limitations, the present trial will use ACTIVATE™ a cognitive computerized program that aims to improve eight different cognitive functions. We will include a sample of children and adolescents with ADHD, and in addition to considering the ratings of clinical symptoms by parents and teachers, we will measure the outcome with an objective, valid and reliable cognitive test battery. The trial is, to our knowledge, the first to examine the effect of cognitive training on the outcome of Cambridge Automated Neurocognitive Test Battery (CANTAB) in children with ADHD.

## Methods/Design

### Objectives

The primary objective is to investigate whether computer training with the games embedded in ACTIVATE™ (http://denmarkstudy2.c8sciences.com/?language=da) has a positive effect on specific cognitive functions. The secondary objectives are to investigate whether there is an effect on ADHD symptoms and functional outcome. Exploratory objectives are to investigate the effects at 12 and 24 weeks after training and to investigate whether younger children benefit more from training than older children.

### Trial sites

Participants are included in three sites in southern Denmark: the Child Psychiatric Departments of Aabenraa (including Augustenborg), Kolding and Odense.

The three sites are part of the same organization, Region of Southern Denmark, and are under the same leadership. All children and adolescent mental health hospitals in Denmark are state-owned, and everyone is eligible to get treatment. Referral from the treating physician or school psychologists is required. No children who are being treated by private practicing child and adolescent psychiatrists will be included in the study. One site (Odense) is part of a university hospital, and the two other sites are part of regional hospitals. Eventual differences between sites will be assessed using data on demographics.

Enrollment of children into the trial is done consecutively throughout the calendar year. The vast majority of children will be enrolled during school year. A few participants will be enrolled during school vacations, but as the intervention is home-based, this will likely not affect the adherence.

### Assessments of eligibility

All children who are newly referred with ADHD symptoms to one of the Child Psychiatric Departments or currently in treatment with ADHD-medication will be invited to participate in the trial and will be offered an individual information session, after which their custodian can give their informed consent. The diagnostic assessment is done in a two-step model: In Step 1 parents, a teacher and children over 11 years of age complete an online questionnaire, including the Strength and Difficulties Questionnaire (SDQ) [58, 59] in conjunction with the psychiatric diagnostic interview Development and Well-being Assessment (DAWBA). The DAWBA is a valid hybrid between a structured and a semi-structured interview for the diagnosis of child and adolescent psychiatric disorders according to both the ICD-10 and DSM-IV [60, 61]. DAWBA’s sensitivity is 92 % in a clinical sample and its specificity is 89 % for all psychiatric diagnoses in a community sample [60]. Parents and teachers answer structured and open-ended questions regarding diagnostic symptoms using the online DAWBA-platform. A child and adolescent psychiatrist rates this information clinically. Children fulfilling diagnostic criteria for ADHD based on this rating of DAWBA proceed to Step 2, which includes a confirmatory clinical interview with parents at the hospital, using the Kiddie-Schedule for Affective Disorders and Schizophrenia (K-SADS, ADHD section) [62]. K-SADS is a semi-structured clinical interview of parents and children and is the most widely used psychometric instrument for the diagnostic investigation of children in clinical research. All children with confirmed ADHD are assessed with Reynolds Intellectual Assessment Scales (RIAS) [63] to ensure that all participants have an IQ above 80. Finally, children are included in the trial if they comply with the following inclusion and exclusion criteria.

### Inclusion criteria

Children are included if the following inclusion criteria are fulfilled: ADHD diagnosis after DAWBA interview [60] and verification with clinical interview K-SADS, ADHD section parent interview [62]; age between 6 and 13 years, both inclusive; the patient has access to a computer and the internet; and informed consent.

### Exclusion criteria

**Fig. 1:**
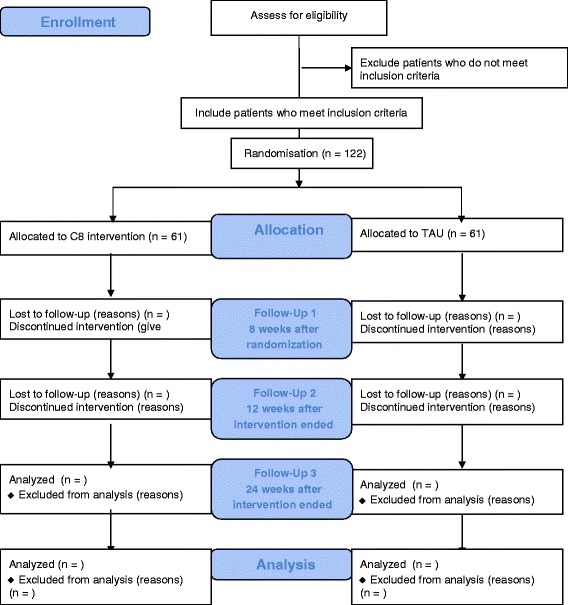
CONSORT 2010 Flow Diagram. Flow diagram of participant enrollment and randomization in the trial

Patients fulfilling any of the following exclusion criteria will not be included: comorbid conduct disorder, autism spectrum disorder, depression or schizophrenia; head injury or verified neurological disease; intelligence quotient (IQ) < 80; motor or perceptual handicaps that prevent computer use; medical condition that requires primary treatment, and no informed consent from custodian (Fig. 1).

### Interventions

Both the intervention group and the control group will receive treatment as usual (TAU). TAU consists of clinical assessment and treatment. Clinical assessment includes intelligence tests, cognitive testing, school observations and parent and teacher questionnaires. TAU may involve psycho-education, parent training, advising the parents and school, and for some children, medication. Parallel to the trial, the participating children will undergo a regular assessment and treatment procedure at the clinic. It is the treating specialist, who is independent of the trial and blind to the child’s randomization status, who will consider possible medical treatment, independent of the child’s participation in the trial. Children in medical treatment are asked not to change their medication dose during the 8 weeks of intervention.

### Intervention group

In addition to TAU, the intervention group will use the computer program ACTIVATE™ (http://denmarkstudy2.c8sciences.com/?language=da). ACTIVATE™ includes an engaging computer program aimed to train multiple cognitive functions simultaneously: sustained attention, working memory, speed of information processing, response inhibition, cognitive flexibility, category formation, pattern recognition and multiple simultaneous attention. ACTIVATE™ consists of three different games: “Catch the ball,” “Butterflies,” and “What comes next.” These are described below.

1. Catch the ball: In this game, there is a ball moving across the computer screen. The child has to use the computer mouse to chase the ball with the arrow on the screen. Every time the ball turns red, the child should put the arrow on the ball, click the mouse, and thus, catch the ball. Every time a child catches a ball, the computer makes a nice sound, and the child gets points. If the child waits too long to click or clicks outside of the ball, s/he will miss the ball, and the computer will make a different sound. The child has to watch the ball all the time and get as many points as possible. When many balls have been caught, the speed will increase. If the child misses some balls, then they will begin to move more slowly, and it will be easier to catch them. The target - red balls or blue balls - will keep changing. The rule is disclosed by looking at the ball on the top of the screen. In the beginning, the child has to catch only the red or blue balls. As the child progresses to higher levels of the game, the rules will begin to change: Initially, the child has to catch a ball when it is the same color as the previous one. Later, the child has to catch a ball if it is a different color than the previous one. Then, two balls bounce across the screen at the same time and have to be watched simultaneously, as all of the rules described before now apply to both balls. Later, three balls - red, blue and green - are introduced, and the child has to catch the blue and red, but never the green balls. “Catch the ball” engages different cognitive functions at the same time: sustained attention, response inhibition and cognitive flexibility. The load on these cognitive functions is increasing during the game and working memory and multiple simultaneous attention are trained at the higher levels of the game.
2. Butterflies: In this game, there are butterflies flying across the screen. Each one carries a sign with a number, a word or a picture on it. The child uses the computer mouse to click on all the signs that have a number or the name of a number. Sometimes the number is spelled out in letters like “t” “w” “o” for 2. These are the targets to look for and click on before the butterfly carries them all the way across the screen. If a child clicks on the wrong butterfly, it falls to the ground. As the child progresses to higher levels, the rules keep changing. The child has to look for signs with letters of the alphabet, animals, plants, furniture and things to take on a vacation. Later, the targets are different kinds of plants with an exception, such as flowers. As the game progresses, the targets change to two different categories, for example, the child has first to click on a picture of food, after that on a picture of clothing, and to continue to go back and forth between the two. This game is mainly engaging the conceptualization of categories and sustained attention on all levels. Some levels also make demands on response inhibition and cognitive flexibility.
3. What comes next: This game trains mainly pattern recognition and, on some higher levels, cognitive flexibility and the use of categories. The child sees some pictures, numbers or shapes in a row that make a pattern or sequence. There is an empty space in the top row. There are some other pictures, numbers or shapes in another set of boxes in a second row below the first. The task is to click on the thing in the second row that goes best in the empty space in the first row, and fits with the pattern on the first row. On the upper right-hand side of the screen, the time allowed to make a choice is displayed. When a child gets questions right, the time remaining to answer the next questions is shortened. Three seconds is the shortest time given to provide an answer. The child graduates and moves on to new levels of the program when enough questions have been answered correctly with only 3 seconds for each question. When a child has worked hard on a level for a long enough time, the computer will move to the next level even if the child was not able to answer the questions within the 3 seconds. The faster the child works, the more points s/he can earn.

In all three games, the child is earning points, which are converted into coins. At the end of all three games, the child comes to a “garden” where s/he can purchase different things. The child can decide to buy things for the garden, such as plants and animals, or a car, a zoo or a house. The child can buy things after each game or can save coins to buy more expensive things later. The games are designed in a manner to be interesting and rewarding for children. All participants are doing the same kind of exercises. All participants start at the same very basic level. Progression and the level of difficulty in the games depend on the child’s performance. Hence, the level of difficulty is therefore dynamically adjusted during the trial, according to the abilities of each child or cognitive phenotype.

Training with ACTIVATE™ is home-based for 40 minutes per day, 6 days per week, for 8 weeks, resulting in a cumulative training of a maximum of 32 hours. ACTIVATE™ records each time the participant logs on and is measuring compliance, time on task and progress. All participants randomized to the intervention group are introduced to the program at the clinic. In case of any problems with the program, the participants can contact the principal investigator. C8 is also providing IT support. Parents are given verbal and written instructions that the child should use a computer with an external mouse (not an iPad or a laptop with an integrated mousepad), that training should be performed in a quiet setting, and that using headphones is mandatory. In addition, the parent are instructed to help the child remember and engage in training and to supervise the child during training sessions, to ensure adherence. We are in touch with parents, and they can contact us any time. Parents are given the instruction to supervise the child and make sure they are doing the training. There were no restrictions on the time of the day the training should be performed. However, we inform parents that most children usually like to do the exercises in the late afternoon or early evening and that parents should ensure that sessions do not conflict with school or family schedules.

### Control group

The control group will only receive treatment as usual.

### Randomization

Randomization is performed centrally by the Copenhagen Trial Unit. Participants are randomized 1:1 to the intervention group or control group. The allocation sequence is computer-generated with a varying block size kept unknown to the investigators, and it is stratified by trial site (“Aabenraa,” “Kolding,” or “Odense”) and use of medication (“yes” or “no”). Allocation is performed by the investigator telephoning the Copenhagen Trial Unit, giving a personal PIN code as well as information about the participant (strata, participant ID number etc.), and the participant is then allocated to an intervention group.

To examine whether the randomization sufficiently reduced the risk of systematic group differences between children in the intervention and the control arm, we will compare the distribution of history of scholar retention events, and pharmacological treatment (dose and type of medication) in the child, mean parental age, socioeconomic status and level of education, and the number of people living at home.

### Blinding

Due to the nature of the trial, it is not possible to blind the participants and their parents. However, we will employ blinding in all other possible areas. Investigators conducting the cognitive testing with CANTAB will be blind to the participants’ group allocation. The statistical analyses will be performed blinded with the two intervention groups coded as, for example, X and Y. The analyses will be presented blinded to the Steering Committee, who will draw two conclusions: one assuming that X is the intervention group and Y is the control group, and one assuming the opposite. After this, the blind will be broken.

To reduce the risk of biasing the rating of outcomes caused by unblinding information on group allocation, we chose an objective computerized primary outcome measure on the CANTAB. Still, the clinicians performing the CANTAB are blinded to group allocation. The treating physicians are not directly connected to the trial and do not assess or provide information on any trial outcomes. Due to regulations by the ethics committee, we were not allowed to inform the treating physician about included children to avoid that influencing the treatment in the outpatient clinic. Hence, as these clinicians were responsible for the treatment as usual (TAU) in both groups, they were also blinded to the group allocation of the child to ensure that this did not affect the TAU.

### Outcomes

For an overview of all outcomes and assessments, please see Table 1.

**Table 1:**
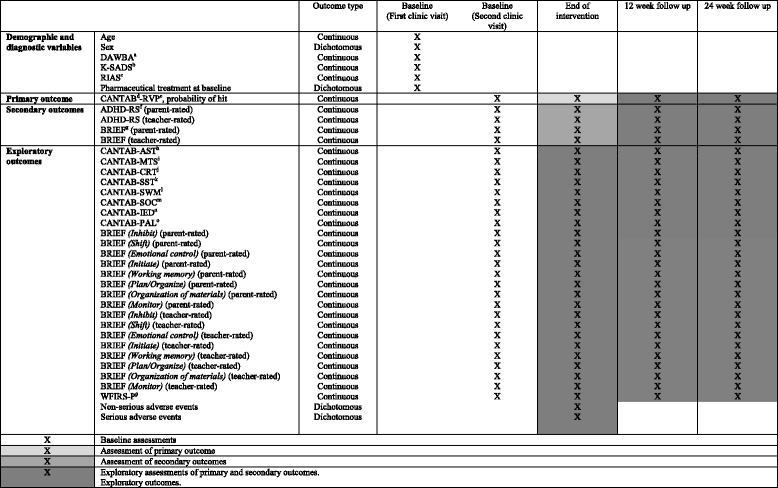
Outcomes and time points for assessment in the trial. Specification of all outcome measures at each time point in the trial

Legend:

^a^Development and Well-being Assessment (DAWBA); ^b^Kiddie-Schedule for Affective Disorders and Schizophrenia (K-SADS); ^c^Reynolds Intellectual Assessment Scales (RIAS); ^d^Cambridge Automated Neurocognitive Test Battery (CANTAB); ^e^Rapid Visual Information Processing (RVP); ^f^Attention Attention Deficit Hyperactivity Disorder-Rating Scale (ADHD-RS); ^g^Behavior Rating Inventory of Executive Functions (BRIEF); ^h^Switching Task (AST); ^i^Match to Sample (MTS); ^j^Choice Reaction Time (CRT); ^k^Stop Signal Task (SST); ^l^Spatial Working Memory (SWM); ^m^Stockings of Cambridge (SOC); ^n^Intra-Extra Dimensional Set Shift (IED); ^o^Paired Associates Learning (PAL); ^p^Weis’s scale of disability-Parent Report (WFIRS-P)

Each CANTAB assessment lasts between 70 and 90 minutes and is collected between 8:30 am and 2:00 pm. While the child is assessed, questionnaire data from the parents are collected. If the child is unable to complete the assessment in one session, the assessment can be split up

Each CANTAB assessment lasts between 70 and 90 minutes. We aim to collect all cognitive assessments between 8:30 am and 2:00 pm to avoid a time of the day that would have an impact on the cognitive performance. While the child is being assessed, the parent questionnaire data are collected. If the child is unable to complete the assessment in one session, the assessment can be split up.

### Primary outcome

CANTAB is a nonverbal computerized cognitive test battery with solid psychometric properties [64–66] (Cambridge Cognition Limited, 2011). The primary outcome is the sustained attention test from the CANTAB: “Rapid Visual Information Processing (RVP) probability of hit,” assessed at the end of the intervention.

### Secondary outcomes

The following secondary outcomes will be assessed at the end of the intervention:

1. Parent-rated ADHD symptoms assessed by ADHD-Rating Scale (ADHD-RS) (parent edition) [67].
2. Teacher-rated ADHD symptoms assessed by ADHD-RS (teacher edition) [67].
3. Parent-rated behavior assessed by Behavior Rating Inventory of Executive Functions (BRIEF) (parent edition) [68].
4. Teacher-rated behavior assessed by BRIEF (teacher-edition) [68].

### Exploratory outcomes

The following exploratory outcomes will be assessed at the end of the intervention:

1. CANTAB Attention Switching Task (AST).
2. CANTAB Match to Sample (MTS).
3. CANTAB Choice Reaction Time (CRT).
4. CANTAB Stop Signal Task (SST).
5. CANTAB Spatial Working Memory (SWM).
6. CANTAB Stockings of Cambridge (SOC).
7. CANTAB Intra-Extra Dimensional Set Shift (IED).
8. CANTAB Paired Associates Learning (PAL).
9. CANTAB RVP Probability of False Alarms.
10. CANTAB RVP Reaction Latency S.D.
11. BRIEF *(Inhibit)* (parent-rated).
12. BRIEF *(Shift)* (parent-rated).
13. BRIEF *(Emotional Control)* (parent-rated).
14. BRIEF *(Initiate)* (parent-rated).
15. BRIEF *(Working Memory)* (parent-rated).
16. BRIEF *(Plan/Organize)* (parent-rated).
17. BRIEF *(Organization of Materials)* (parent-rated).
18. BRIEF *(Monitor)* (parent-rated).
19. BRIEF *(Inhibit)* (teacher-rated).
20. BRIEF *(Shift)* (teacher-rated).
21. BRIEF *(Emotional Control)* (teacher-rated).
22. BRIEF *(Initiate)* (teacher-rated).
23. BRIEF *(Working Memory)* (teacher-rated).
24. BRIEF *(Plan/Organize)* (teacher-rated).
25. BRIEF *(Organization of Materials)* (teacher-rated).
26. BRIEF *(Monitor)* (teacher-rated).
27. Weis’s scale of disability-Parent Report (WFIRS-P) (Weis et al., 2005).
28. Non-serious adverse events.
29. Serious adverse events.

**Fig. 2:**
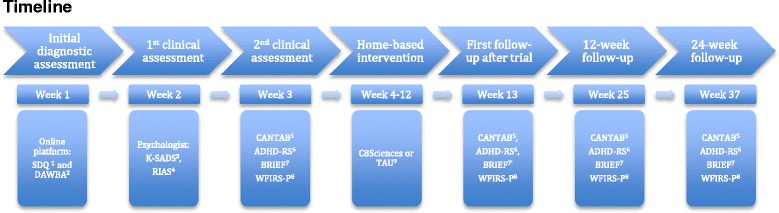
Timeline of the study enrollment. Timeline of participant assessments in the trial. 1) SDQ, Strength and Difficulties Questionnaire; 2) DAWBA, Development and Well-being Assessment; 3) K-SADS, Kiddie-Schedule for Affective Disorders and Schizophrenia; 4) RIAS, Reynolds Intellectual Assessment Scales; 5) CANTAB, Cambridge Automated Neurocognitive Test Battery; 6) ADHD-RS, Attention Deficit Hyperactivity Disorder-Rating Scale; 7) BRIEF, Behavior Rating Inventory of Executive Functions; 8) WFIRS-P, Weis’s scale of disability-Parent Report; and 9) TAU, treatment as usual

Further, all outcomes will be assessed again 12 and 24 weeks after the end of the intervention (Fig. 2).

### Ethical issues

This trial is being conducted in accordance with the protocol approved by the Danish Data Protection Agency (ID.nr. 2008-58-0035) and the Regional Scientific Ethical Committees for Southern Denmark (nr. S20120096). The protocol is in accordance with the latest version of the Declaration of Helsinki. No significant deviation from the protocol will be implemented without prior review and approval by the regulatory authorities unless it may be necessary to eliminate an immediate hazard to the trial participants. In this case, the deviation will be reported to the regulatory authorities as quickly as possible.

A child can participate in the trial if the written consent of both parents is obtained. The patients’ treatment as usual will not be affected, including the use of medications, by their participation in this trial. When testing with CANTAB, patients will be asked to postpone any ADHD medication for 24 hours before testing, when medical treatment can affect efficacy measures. Trial participants will receive a gift certificate worth DKK 400 for participation. Transportation costs will be reimbursed.

The processing of personal data will be respected. There are no known risks associated with the use of computerized cognitive training. The method has been tested in many studies with patients with schizophrenia (see reviews [69, 70]) and in children with ADHD [38–40, 53]. No adverse events have been reported.

### Discontinuation and withdrawal

Although parents may have agreed to participate, they can always withdraw their child from the trial without further explanation. Pulling a child out of the trial, will not affects his or her further treatment. Patients can choose to stop at any time in the trial. Patients who were randomized will be included in the intention-to treat analyses unless they completely withdraw consent. In this case, all data regarding this participant will be deleted.

### Statistical plan and data analysis

#### Sample size

We are planning a trial of a continuous response variable, “CANTAB RVP probability of hit,” from an independent control, and experimental participants will be allocated at a 1:1 ratio. In a pilot project (Bikic et al., unpublished data), adolescents with ADHD played a set of games from Scientific Brain Management for 7 weeks. Here, the “CANTAB RVP probability of hit” was normally distributed, with a standard deviation (SD) of 0.22 points. If the true difference in the experimental and control mean is 0.13 points, we will need to include 61 experimental participants and 61 control participants to be able to reject the null hypothesis that the population means of the experimental and control groups are equal with a probability (power) of 90 %. The type I error probability associated with this test of this null hypothesis is 5 %. We will thus include 122 participants in total.

#### Power

Assuming the minimal relevant difference is 0.5 SD for all the secondary outcomes and the significance level of the various tests of Hommel’s procedure is within the range of alpha = 0.05 and 0.05/4 = 0.0125 and the sample size is 122, the power of the individual tests will range between 59 % and 78 % (Table 2). A power of 78 % is judged to be reasonable, whereas a power of 59 % is insufficient.

**Table 2:** Power estimations for the secondary outcome measures ADHD-RS and BRIEF

Outcome	Minimal relevant difference	SD^a^	Sample size	Power assuming an alpha of 1.25 %	Power assuming an alpha of 5 %
ADHD-RS^b^ (parents-assessed)	5 points	10 points	122	59 %	78 %
ADHD-RS (teacher-assessed)	5 points	10 points	122	59 %	78 %
BRIEF^c^ (parents-assessed)	0.25 points	0.5 points	122	59 %	78 %
BRIEF (teacher-assessed)	0.25 points	0.5 points	122	59 %	78 %

Legend:

^a^SD, standard deviation

^b^ADHD-RS, attention deficit hyperactivity disorder rating scale. Minimal relevant difference and SD calculated from a previous pilot project (Bikic et al. unpublished data)

^c^BRIEF, Behavior Rating Inventory of Executive Functions. Minimal relevant difference and SD calculated from the BRIEF professional manual [68]

#### Multiplicity and significance

For all outcomes, we will present the test statistic and the corresponding *P* values for exploratory purposes.

The purpose of the analysis of the secondary outcomes is to make additional claims of treatment benefits in addition to those already established by the analysis of the primary outcome. Consequently, multiplicity adjustments are needed. Multiplicity adjustments generally require a strong control of the familywise error rate. With regard to this, a useful approach is the gatekeeping approach [71], which we will apply in this trial.

There is one primary and four secondary outcomes. Thus, the primary outcome will be the gatekeeper of the family of secondary outcomes. The sample size has been estimated using a risk of type I error (alpha) of 0.05. The primary outcome will consequently be analyzed and interpreted according to a two-sided significance level of *P* ≤ 0.05. Thus, if P of the test of the primary outcome is ≤ 0.05, the primary outcome is assessed as statistically significant. In this case, we will use Hommel’s procedure, which is uniformly more powerful than the Holm as well as the Hochberg adjustment procedures. This means that the alpha of 0.05 can be transferred to the secondary outcomes that will be tested in a pre-specified sequence at the 0.05 level of significance (see sequence in Table 2). The approach requires that as soon as the *P* value of a test is > 0.05, the null hypotheses of the remaining secondary outcomes are accepted regardless of the test statistics.

If *P* of the test of the primary outcome is > 0.05, the primary outcome is assessed as statistically insignificant. Consequently, the trial result is insignificant, and all the null hypotheses of the four secondary outcomes will be accepted regardless of the test statistic.

All exploratory outcomes and exploratory analyses of the primary and secondary outcomes will likewise be subject to statistical tests. However, if P of the test is ≤ 0.05, the outcome will not be assessed as statistically significant due to multiplicity and the increased risk of a type I error. Likewise, if P > 0.05, we cannot assess the outcome as statistically insignificant due to a potential lack of power. All exploratory analyses will thus be strictly hypothesis generating.

#### Analytical model

For the analysis of the continuous outcomes, structural equation models (for example, “proc calis” in SAS 9.3) including the direct maximum likelihood method (full information likelihood) will be used (see section on missing values). If the assumptions of a regression analysis are not fulfilled, a non-parametric test will be used (van Elteren’s test with stratification by one variable “center”). For dichotomous outcomes, we will use logistic regression to compare the results in the two groups.

#### Adjustments

All analyses will be adjusted for the stratification variables (“center” and “pharmaceutical treatment at baseline”), and the outcome variable value will be assessed at baseline.

#### Missing values

In the analysis of the continuous variables, structural equation models (for example, “proc calis” in SAS 9.3) that include the direct maximum likelihood method (full information likelihood) will be used. Applying this method implies that unbiased estimates of the regression parameters will be obtained as long as the values are only missing at random. To improve the efficiency, all auxiliary variables present among the outcomes will be added to the model. An auxiliary variable is defined as a variable not in the analytical model but correlated (defined as |r| > 0.40) with one or more variables that 1) have missing values, and 2) are included in the analytical model. Thus, the auxiliary variables included may vary from one regression equation to the next one.

#### Sensitivity analyses

Best-worst and worst-best sensitivity analyses of the primary outcome will be done. Here, missing values in one intervention group are imputed by the minimum value found in the total material (“best case”), and missing values in the other group are imputed by the maximum value found in the total material (“worst case”) and vice versa. The range of the estimates of the two regression parameters of the intervention indicator will convey an impression of the bias one may expect if values are missing not at random.

#### Per-protocol analyses

For the primary and secondary outcomes, we will use exploratory analyses to perform per-protocol analyses. Participants will be included in the intervention group, if they have complied with at least 20 out of the 48 scheduled computer training sessions. Participants will be included in the control group if they have not attended any computer training sessions.

#### Subgroup analyses

For the primary and secondary outcomes, we will perform subgroup analyses according to age. We will divide the participants into two age groups of children aged 6 to 9 years or 10 to 13 years. We will perform a test of interaction to assess whether the effect of the intervention is different among the younger children compared with the older children. If *P* of the test of interaction is ≤ 0.05, we will present separate estimates for the two subgroups. As the randomization procedure was not stratified by age and we most likely will have reduced power for this analysis, the result is exploratory and strictly hypothesis generating.

#### Sequential analysis

As the recruitment in the trial until present has been slower than anticipated, we may face a scenario where we have to end recruitment before the sample size of 122 participants has been met. In this case, we plan to perform sequential analysis to assess the results of significance testing, taking sparse data and into consideration [72]. We will for the primary outcome, CANTAB-RVP, use a minimal clinically relevant difference of 0.13 and a variance of 0.0484 (corresponding to a SD of 0.22). For the secondary outcomes, we will use minimally relevant differences of 5 points and a variance of 100 (corresponding to a SD of 10 points) for both AHDH-RS assessments and a minimal clinical relevance of 0.25 points and variance of 0.25 (corresponding to a SD of 0.5 points) for both BRIEF assessments. For all outcomes, we will use a type I error of 5 %, and a type II error of 10 %. We will use the trial sequential analysis program for these analyses (http://ctu.dk/tsa/) [73–76].

### Discussion of clinical relevance

If the trial results indicate that this intervention reduces specific cognitive deficits in children with ADHD without causing any adverse reactions effects, our interpretation will be that the intervention can be an important part of a treatment plan as cognitive dysfunctions are very common in children with ADHD. Furthermore, if we find improvement in the BRIEF measures and ADHD-RS, this would suggest that the effects of the intervention could be generalized to behavior in an everyday setting.

### Monitoring of patient compliance issues

The intervention group compliance will be monitored via the computer program that records patients log on, which games they have played, and for how long.

### Financial support

Aida Bikic is the initiator of the trial and the investigator psychologist, research coordinator and PhD student. Participants are being randomized in the Child and Adolescent Psychiatric setting of Augustenborg and Aabenraa, Odense and Kolding. The trial has received grants from Region of Southern Denmark Psychiatry Research, The Region of Southern Denmark’s PhD pool, Child and Adolescent Psychiatric Department Aabenraa, TrygFonden (J.nr. 7-12-1137) and the University of Southern Denmark. C8 Sciences allowed us to use the ACTIVATE™ program at no charge in this trial. None of the funders has any role in the development of the trial design, trial conduct or trial reporting.

## Discussion

This trial is a multicenter, randomized clinical superiority trial investigating the effect of a home-based 8-week intervention for children with ADHD, using a computerized cognitive training program, ACTIVATE™. ACTIVATE™ was designed to enhance a broad range of cognitive functions. The trial has several strengths: it is conducted with adequate generation of allocation sequence; adequate allocation concealment; adequate blinding wherever possible; adequate reporting of all relevant outcomes; adequate handling of incomplete outcome data; and has no for-profit bias [77–80]. The trial results will offer new and valuable contributions to the field of cognitive training in children with ADHD.

The trial also has some limitations. Due to the nature of the trial, it is not possible to blind the participants, their parents, or their teachers. A “sham” intervention for the control group was considered. However, in order to introduce an active control intervention that would function as a true placebo, we needed to be sure that this intervention had no beneficial or harmful effects, which is difficult to document. Furthermore, a placebo-training program would be somewhat boring in order to have no training effect. This would potentially cause problems with low adherence in the control group and reveal group allocation. We consequently chose a “treatment as usual” control group, thereby accepting the risk of bias regarding the blinding that this entails.

We included both drug-naïve children and children receiving pharmaceutical therapy in the trial. As the randomization procedure is stratified for this variable, it is not expected to influence the trial results. Furthermore, all children are required to be free of medication 24 hours before cognitive testing in order to influence the results as little as possible. All patients are required to perform cognitive assessments at four time points without medication. This may present a potential threat of bias in terms that patients, who are not able to function without medication for 24 hours prior to the testing, could choose not to participate in the study. In this case, some of the more severe cases with ADHD might not be represented in the study sample. Overall, inclusion of children regardless of pharmaceutical treatment status is expected to increase the external validity of the trial results.

We do not consider dropout of medical treatment during the trial a threatening issue. In Denmark, ADHD assessments, diagnostic procedures and initiation of ADHD-medications is restricted to specialists of child and adolescent psychiatry, and general practitioners are not allowed to initiate this treatment [81, 82]. This result in a lower prevalence of children and adolescents treated with ADHD-medications (prevalence in 2010 was only 1.5 % [83], compared to most other Europeans countries and certainly to most parts of the USA, and probably to a less negative public attitude toward medication. Adherence to medication in Denmark is likely higher than in many other countries. The study protocol requires medicated children to stay on medication during the intervention period. Parents are encouraged to continue the children’s medication, if they are on it at time of inclusion.

## Trial status

The first participant was included and randomized in March 2013. Recruitment is currently ongoing.

## Abbreviations

ADHD: Attention Deficit Hyperactivity Disorder
ADHD-RS: Attention Deficit Hyperactivity-Rating Scale
AST: Attention switching task
BRIEF: Behavior Rating Inventory of Executive Functions
CANTAB: Cambridge Automated Neurocognitive Test Battery
CRT: Choice reaction time
DAWBA: Development and Well-being Assessment
IED: Intra-Extra Dimensional Set Shift
K-SADS: Kiddie-Schedule for Affective Disorders and Schizophrenia
MTS: Match to sample
PAL: Paired associates learning
RIAS: Reynolds Intellectual Assessment Scales
RVP: Rapid visual information processing
SWM: Spatial working memory
SOC: Stockings of Cambridge
SST: Stop Signal Task
SDQ: Strength and Difficulties Questionnaire
TAU: Treatment as usual
WFIRS-P: Weis’s scale of disability-Parent Report
